# Supplementary material for: A non-linear system patterns Rab5 GTPase on the membrane
Source: eLife. 2020 Jun 8;9:e54434. doi: 10.7554/eLife.54434 (PMC7279886; doi:10.7554/eLife.54434)
Supplement: Transparent reporting form [file elife-54434-transrepform.docx]

***eLife’s* transparent reporting form**

We encourage authors to provide detailed information *within their submission* to facilitate the interpretation and replication of experiments. Authors can upload supporting documentation to indicate the use of appropriate reporting guidelines for health-related research (see [EQUATOR Network](about:blank)), life science research (see the [BioSharing Information Resource](about:blank)), or the [ARRIVE guidelines](about:blank) for reporting work involving animal research. Where applicable, authors should refer to any relevant reporting standards documents in this form.

If you have any questions, please consult our Journal Policies and/or contact us: [editorial@elifesciences.org](about:blank).

**Sample-size estimation**

- You should state whether an appropriate sample size was computed when the study was being designed
- You should state the statistical method of sample size computation and any required assumptions
- If no explicit power analysis was used, you should describe how you decided what sample (replicate) size (number) to use

Please outline where this information can be found within the submission (e.g., sections or figure legends), or explain why this information doesn’t apply to your submission:

No appropriate sample size computation was performed. Experiments were done minimally in triplicate (biological replicates on different days with different supported bilayer preparations), with at least two different liposome preparations.

**Replicates**

- You should report how often each experiment was performed
- You should include a definition of biological versus technical replication
- The data obtained should be provided and sufficient information should be provided to indicate the number of independent biological and/or technical replicates
- If you encountered any outliers, you should describe how these were handled
- Criteria for exclusion/inclusion of data should be clearly stated
- High-throughput sequence data should be uploaded before submission, with a private link for reviewers provided (these are available from both GEO and ArrayExpress)

Please outline where this information can be found within the submission (e.g., sections or figure legends), or explain why this information doesn’t apply to your submission:

Tables 1, 2 & 3 show sample sizes for experiments presented in Figures 2, 3, 4 & 6 as well as Supplemental Figures. Minimum 40 beads were imaged, per condition, however not all beads imaged were reconstructable and as such could not be characterized (i.e. bead shape defects, beads too dense). Where n < 30 (e.g Table 2), non-reconstructable beads were checked manually to see whether the total population was reflected by the reconstructable population.

Segmented domains that resulted from membrane defects (marked by negative correlation between 488/660 channels) were excluded from the analysis.

**Statistical reporting**

- Statistical analysis methods should be described and justified
- Raw data should be presented in figures whenever informative to do so (typically when N per group is less than 10)
- For each experiment, you should identify the statistical tests used, exact values of N, definitions of center, methods of multiple test correction, and dispersion and precision measures (e.g., mean, median, SD, SEM, confidence intervals; and, for the major substantive results, a measure of effect size (e.g., Pearson's r, Cohen's d)
- Report exact p-values wherever possible alongside the summary statistics and 95% confidence intervals. These should be reported for all key questions and not only when the p-value is less than 0.05.

Please outline where this information can be found within the submission (e.g., sections or figure legends), or explain why this information doesn’t apply to your submission:

Unpaired t-tests were performed in Figures 2, 3, 4, 5 & 6, as well as Supplemental Figure 3. Statistical significance is noted on graphs and p-values reported in the Figure Legends. Where samples were not statistically significant, no p-value is reported. Presentation of summary statistics in box and whisker plots (Figures 2, 3, 4, 5 & 6) is described in Methods section ‘Image Analysis’.

(For large datasets, or papers with a very large number of statistical tests, you may upload a single table file with tests, Ns, etc., with reference to sections in the manuscript.)

**Group allocation**

- Indicate how samples were allocated into experimental groups (in the case of clinical studies, please specify allocation to treatment method); if randomization was used, please also state if restricted randomization was applied
- Indicate if masking was used during group allocation, data collection and/or data analysis

Please outline where this information can be found within the submission (e.g., sections or figure legends), or explain why this information doesn’t apply to your submission:

Group allocation was not considered during experimental design, however bead samples were only used if membrane quality was sufficient. For imaging, fields of view were chosen randomly, based on membrane signal, not protein signal.

**Additional data files (“source data”)**

- We encourage you to upload relevant additional data files, such as numerical data that are represented as a graph in a figure, or as a summary table
- Where provided, these should be in the most useful format, and they can be uploaded as “Source data” files linked to a main figure or table
- Include model definition files including the full list of parameters used
- Include code used for data analysis (e.g., R, MatLab)
- Avoid stating that data files are “available upon request”

Please indicate the figures or tables for which source data files have been provided:

Excel file “Domain-quantification” contains source data for graphs in Figures 2, 3, 4 & 6.
